# Psychiatric services in Jaipur: Past and present

**DOI:** 10.4103/0019-5545.58909

**Published:** 2010

**Authors:** Shiv Gautam

**Affiliations:** Department of Psychiatry Psychiatric Center, and S.M.S. Medical College, Jaipur, India

## ERA OF ASYLUMS (1856-1944)

To view the changing perspective of Psychiatry in Rajasthan, we must begin with the story of establishment of lunatic asylums in Rajputana states. It was after the treaty of alliance signed between the native states of Rajputana and the East India Company (1803-1818) that each native state created/established an asylum for detention and care of the lunatics. Initially the lunatics were seen in dispensaries and detained in jails tied with chains. An asylum outside the jail was first established in 1881 in Jaipur state. Ajmer had an asylum inside jail in 1865 (Hendley 1900). Forty unfortunate lunatics were incarcerated inside ‘cells’ in this asylum, which was situated near Ghatgate (Jaipur). In year 1894, another enclosure in a walled garden near Central Telegraph Office, Jaipur in the southern part of the city was used as asylum by Jaipur state. Here the lunatics were left to enjoy the Open Court Yard and a ‘Baradaree’, no cots were provided but the atmosphere was more humane and sympathetic towards the unfortunate lunatics who were confined in this asylum. Food and protection were provided by the jail department till 1922, when this asylum was shifted to a Dharamshala situated near Chandpole Gate opposite the present Janana Hospital. Here the lunatics were again put in cells. The medical supervision was provided by the part-time doctors from the jail department. Administration, watch and ward of asylum remained under the jail department till year 1943 (Annual Administrative Reports of Jaipur State, 1894-1943).

In 1943, Jaipur state enacted Jaipur Lunacy Act for detention and treatment of lunatics; the criminal procedure code 1926. Jaipur State was amended for the detention and care of the criminal lunatics 1943 (C.P.U. 1943). From the year 1944, the Mental Hospital Jaipur (Lunatic Asylum) started running as an independent hospital under Medical Department of Jaipur State. Till 1922, the control of lunatic asylum was placed under the Rajputana Agency, but after 1922 the administration of lunatic asylum was transferred to Jaipur Durbar. The annual returns from these asylums show a high mortality of lunatics admitted there in, on account of diarrhea, dysentery and fever. Under orders from Jaipur Durbar, the lunatics could be released from asylums for the care of relatives and friends if assured for kind sympathetic care and protection from doing harm to others. All lunatics were admitted under court orders.

The discipline of psychiatry is rather a new specialty of medical science. Though understanding of human mind has existed from times immemorial in different philosophical and religious schools, in the middle ages, due to lack of knowledge, superstitions like evil spirits dominated the explanation for various mental disorders. It is only in the late 19^th^ century that psychoanalytic explanations came in and the last four decades have seen tremendous scientific advancements in understanding of human behavior through biopsychosocial research. The treatment of mental disorders has also been revolutionized in the last four decades and as a result to this the specialty is now a very fast growing subject.

As a result of research in mental health, now intricate complexities of the human mind have been understood in terms of neuroelectrical and neurochemical changes occurring in the brain. Specific change occurring with different mood states has been identified and an intervention to bring desired changes in human behavior is now possible. Mental illnesses once thought incurable are now treatable. Mental illness is now considered like any other illness and there is no reason that it should be concealed, and like patients with physical disorder the mentally ill after treatment can be rehabilitated and made useful citizens.

## PSYCHIATRIC CENTER, JAIPUR

Psychiatric Centre, Jaipur is the premier institute of the state for treatment and care of mentally ill. Founded in 1952, the institution has completed 53 years of its existence. Like the discipline of Psychiatry, the initial development was rather slow. But after its recognition as a postgraduate teaching department of the medical college in the year 1980, development has been seen in certain areas of its growth.

It is one of the six attached hospitals of S.M.S. Medical College, Jaipur. The center was started with a bed strength of 180 with an outpatient attendance of 190 per annum and annual turnover of 105 indoor patients in the year 1952. At present the hospital has a total bed strength of 320 (280 in door patients, 20 in emergency ward and 20 in de-addiction ward). The hospital has an annual turnover of average 4000 indoor patients and an outpatients' attendance of nearly 40,000 per annum.

The catchment area of the hospital is whole of Rajasthan and adjacent states of U.P., Madhya Pradesh, Haryana, Punjab and State of Delhi. The admission facilities in the center are free to every person irrespective of the state. The medicines are distributed free of cost to all the indoor as well as outdoor patients.

A 20-bedded emergency ward was started in the year 1981 for the patients requiring urgent care and management. The admission facility in the emergency ward is available round the clock. A separate 12-bedded de-addiction ward was also started in the year 1986 to meet the special needs of the drug-addicts.

The outpatients department of the Center is run by consultants, civil assistant surgeons and postgraduate students. At present there are three Associate Professors, six Assistant Professors, five CAS and nine postgraduate students. There are posts for two clinical psychologists and two psychiatric social workers, who collaborate with the professionals for the psychological testing and psychosocial therapies.

The Center has facilities for routine biochemical and pathological testing. Special investigations like EEG, ECG, X-ray, serum lithium estimation and CAT SCAN etc. are either available at the Center or at SMS Hospital. A Bio-feedback apparatus has been added to the department in the year 1996-1997.

There is a separate rehabilitation center for occupational therapy, where patients are given opportunity to work and learn some skills according to their aptitude and therapeutic needs, patients are being involved in the work of carpentry, canning, gardening, painting, in kitchen work, making paper bags for the distribution of medicines in the outpatients department. The rehabilitation therapy department needs to be better equipped to be helpful in the long-term rehabilitation of patients.

A regular General Hospital Psychiatric Clinic is being run at S.M.S. Hospital by the consultants and senior postgraduate students from the department. A 20-bedded de-addiction ward has been started at SMS Hospital. It meets the needs of those drug-addicts who do not want to get admitted at the Psychiatric Center. The need for such a facility was long felt.

## TEACHING AND RESEARCH ACTIVITIES

The postgraduate course in psychiatry was started at Jaipur in the year 1979. So far 80 postgraduates have passed out from the department and three new students are registered every year for the M.D. Degree in Psychiatry. Most of the students who have passed out from this center are working in different positions at various places in the state, outside the state in India and abroad.

The department is imparting training to both undergraduates and postgraduate student. The training involves theory classes as well as clinical work in outpatient departments. The postgraduate teaching also includes regular case conferences, seminars, tutorials and review of research articles from journals, etc. The postgraduate students also undergo training in Neurology, Neurosurgery, Child Psychiatry, De-addiction Center and Neuroradiology.

The institution as a postgraduate and undergraduate teaching department has established itself as one of the important training center in North India. Professors of these departments act as external examiners in about 8-10 universities of the country and this institution has been recognized as an National Board of Examination Center for the National Academy of Medical Science (Dip. NB). Students from all over the country come here for writing the examinations.

The students of B.Sc. Nursing come to the center for their practical training in psychiatry. The students of Psychology and General Nursing, from within the state and outside, visit the center for the purpose of teaching and clinical work.

The center has also been recognized as one of the five regional center for the implementation of National Mental Health Program (NMHP) of India in the year 1996-1997. Two training programs for the trainers have been organized in the same year, wherein doctors from all the six medical colleges and five district headquarters received the training. Sikar was identified as the district for the implementation of District Mental Health Program under NMHP. Under the training of trainers program supported by Government of India, Psychiatrists from Rajasthan, Punjab, Haryana and Delhi were trained.

The department also organizes de-addiction camps in the community with the co-operations of voluntary organization. The center was also given the responsibility of training of doctors from PHC's in de-addiction management.

School Mental Health Program has also been initiated by the department and an awareness building program for mental illnesses and drug abuse prevention in school going children has been initiated. A training program for the school teachers was also organized for their orientation and role as counselors in emotional and behavioral problems of school children.

## DEPARTMENTAL RESEARCH

There are ample opportunities for research in Psychiatry and related disciplines in behavioral sciences at the center. The postgraduate students through their work on the dissertations are also taught the basic principle of research methodology.

The center has been actively involved in psychiatric research. Two projects supported by Indian Council of Medical Research (ICMR), one projects supported by Mental Health Foundation, United Kingdom (Jaipur-London project) apart from the regular departmental research have been completed. The center has also participated in the World Health Organization (WHO) field trials for International Classification of Diseases, putting this department on the world map of Mental Health.

Some projects and papers frequently quoted/cited are:-

1. Gehlot PS, Gautam S, Gupta SD. ICMR Epidemiological study of mental illness in a migrant urban population (1981-1983).
2. Gautam S. ICMR Project on Primary Mental Health Care comparative study of efficacy of Training Material and Development of record system (1983-1984).
3. Gautam S, Nijhawan M. Burden on families of schizophrenic, cancer and chronic lung disease patients. Indian J Psychiatry 1984;26:156-9.
4. Gautam S. Development and Evaluation of Training Programmes for Primary Mental Health care. Indian J Psychiatry 1985;27:51-62. Received “Bhagat Award” for best research paper by scientist under 35 years at 37^th^ Annual Conference of Indian Psychiatric Society at Vishakhapatnam Jan. 1985.
5. Gautam S, Nijhawan M, Kamal P. Standardisation of Hindi version of Goldbergs, General Health Questionnaire. Indian J Psychiatry 1987;29:63-6.
6. Gautam S, Nijhawan M. Communicating with cancer patients. Br J Psychiatry 1987;150:760-4: Included in Year Book of Psychiatry and Applied Mental Health 1989.
7. Gautam S. Tilak Venkoba Rao Oration (1989): Post Partum Psy. Syndromes: Are they biologically determied? Indian J Psychiatry 1989;31:31-42.
8. Batra L, Gautam S. Psychiatric morbidity and personality profile in divorce seeking couples. Indian J Psychiatry 1995;37:179-85.
9. Gautam S, Batra L. Sexual behaviour and dysfunction in divorce seeking couples. Indian J Psychiatry 1996;38:109-16 (Marfatia Award Paper of IPS.)
10. Gaita S, Agarwal R, Sharma H. Election: A stressful life event. Indian J Psychiatry 1997;39:329-32.
11. Gautam S, Guptal D, Batra L, Sharma H, Khandelwal R, Pant A. Psychiatric morbidity among victims of bomb blast. Indian J Psychiatry 1999;41:41-5 (Frequently quoted in Disaster Management).
12. Gautam S. IPS Presidential address: Mental Health in ancient India and its relevance to modern psychiatry. Indian J Psychiatry 1999;1:5-18.

Apart from this many other research activities are going on in the center. Nearly 200 scientific research papers have been published and presented by the members of the faculty in National and International journals and conferences. Author has received various prestigious awards namely Bhagwat Award, Marfatia Award, Tilak Venkoba Rao Oration Award, Poona Psychiatric Association Award at National Level and International New Horizon Award for work in de-addiction and drug awareness.

## IMPORTANT CONFERENCES, SEMINARS AND WORKSHOP ORGANIZED BY THE DEPARTMENT OF PSYCHIATRY

1. World Psychiatric Association Symposia and Regional conference 1986, 1998.
2. National Conferences of Indian Psychiatric Society 2003.
3. North Zone Annual Conference of Indian Psychiatric Society 2003.
4. National Mid-term CME of Indian Psychiatric Society (Tradition was initiated from Jaipur) 1990 and 1996.
5. National workshop on ‘Psychiatry Law and Society’ of Indian Psychiatric Society 1989.
6. National Workshop on ‘Clinical Practice Guidelines for Psychiatrists in India’ of Indian Psychiatric Society 2004 and 2005.
7. Training of Trainers Program supported by Government of India organized in 1996, 1997. Trainees included psychiatrists from Rajasthan, Punjab, Haryana and Delhi.
8. Several State Conferences and Workshops of Rajasthan Psychiatric Society have been organized (1984, 2003, 2005).
9. National Mental Health Program: More than 500 doctors, MPW and community leaders have been trained by this department under National Mental Health Program.

## HISTROCIAL DEVELOPMENTAL LAND MARKS OF PSYCHIATRIC CENTER, JAIPUR

Established in 1952 as UG Department of Psychiatry under Department of Medicine, S.M.S. Medical College, Jaipur, the department was running inpatient, Outpatient department and undergraduate medical education up to 1979.

**Table:** Development of services

Year	Suptdt. and HOD	Activity
1980	Dr. P.S. Gehlot	Recognized as PG teaching department of psychiatry. General Hospital Psychiatry OPD was started once gradually converted in regular OPD at SMS Hospital 7 days a week, 50 to 60 patients avail services every day.
1981	Dr. P.S. Gehlot	Emergency psychiatry ward started at Psychiatric Center, Jaipur.
1987	Dr. J.N. Vyas	De-addiction ward started at Psychiatric Center, Jaipur.
1987	Dr. J.N. Vyas	Well-equipped Seminar Hall at Psychiatric Center was furnished.
1998	Dr. Shiv Gautam	De-addiction ward was started at SMS Hospital.
1998	Dr. Shiv Gautam	17 staff quarters in hospital campus were constructed.
1998	Dr. Shiv Gautam	Behavior therapy laboratory was started along with bio-feedback facility at psychiatric center, Jaipur.
1999	Dr. Shiv Gautam	National Mental health Program with support of Govt. of India.
2003	Dr. Shiv Gautam	New establishment section and research wing with Internet Facilities for PG students were started with support of MP/LAD funds/Research projects.
2004	Dr. Shiv Gautam	A pilot project at Sikar was completed.
2004	Dr. Shiv Gautam	A modern new OPD block came to functioning.
2004	Dr. Shiv Gautam	Child and adolescent psychiatry block came in functioning with support of MP/LAD funds.
2004	Dr. Shiv Gautam	Psychiatric rehabilitation center started at Psychiatric Center, Jaipur.
2004	Dr. Shiv Gautam	Well-equipped biochemistry laboratory was started in OPD.
2004	Dr. Shiv Gautam	Modern EEG with video facility was started in OPD.
2004	Dr. Shiv Gautam	New laundry and auto machines were added for improved patient care.
2005	Dr. Shiv Gautam	Yoga and meditation facilities were introduced in de-addiction ward.
2005	Dr. Shiv Gautam	Upgradation of ultra modern de-addiction ward with semi ICU ward at S.M.S. hospital.
2005	Dr. Shiv Gautam	Departmental library was upgraded with full time staff.
2006	Dr. Shiv Gautam	Hospital kitchen was modernized.
2007	Dr. Shiv Gautam	12 new staff quarters in hospital campus were constructed.
2008	Dr. Shiv Gautam	Geriatric psychiatry wing.
2008	Dr. Shiv Gautam	Construction of 12 No. of family psychiatric ward, three dining halls and NMHP trainee's hostel is in progress likely to be completed by last week of April 2009.

Important dignitaries who visited the department

- Dr. N.N. Wig, Professor Dmirtus, P.G.I. Chandigarh and Advisor to W.H.O. Regional Center, Alexandria (in 1979 for the inspection of department).
- Linda B. Johnson, President - American Psychiatric Association (APA) in 1986.
- Prof. Costa Stefanis, (Athens, Greece) President, World Psychiatric Association (W.P.A.) in 1986.
- Dr. J.S. Bajaj, Member Planning Commission - Govt. of India in 1986.
- Prof. Schulsinger, Secretary General (W.P.A.) in 1986.
- Prof. Abdul Rasheed Chaudhary, President, Pakistan Psychiatric Association in 1986.
- Late Justice B.P. Beri, Chairman - Beri Commission and Former Chief Justice of Rajasthan High Court in 1989.
- Dr. Robert Passnav, President, American Psychiatric Association in 1987.
- Dr. Melvin Sabshin, Medical Director, American Psychiatric Association in 1986.
- Prof. G.N.N.R. Reddy, Director (NIMHANS, Bangalore) in 1987-1989.
- Dr. S.M. Channabasavanna, Vice Chancellor (NIMHANS) in 1987
- Dr. Norman Sartorius, President World Psychiatric Association in 1997 and 1998.
- Prof. R. Srinivasmurthy, Regional Advisor (WHO) and Former Prof. and Head (NIMHANS), Bangalore (1986-1987)(Served as faculty for NMHP in two training programs.
- Prof. Ahmed Okasha, President (WPA) visited in 1998.
- Prof. Juan Mezzich - USA, Current President (WPA) visited in 1998
- Prof. Mario Maz (Germany)-President Elect. (WPA) visited in 1998
- Padamshree Dr. Mohan Agashe-Film Actor and Professor Head Psychiatry, Pune in 1998
- Prof. H.M. Gadhvi, Senior consultant, London U.K. visiting Professor-1999-2006.

Superintendents, HOD and Professors of Psychiatric Center and their Tenure

1. Dr. B.K. Vyas 24.07.1967 to 31.12.1978
2. Dr. P.S. Gehlot 01.01.1979 to 30.04.1984
3. Dr. J.N. Vyas 28.05.1984 to 03.10.1989
4. Dr. G.B. Advani 04.10.1989 to 18.01.1990
5. Dr. J.N. Vyas 19.01.1990 to 05.05.1990
6. Dr. G.B. Advani 06.05.1990 to 23.02.1996
7. Dr. Shiv Gautam 01.03.1996 to 25.07.2000
8. Dr. L.N. Gupta 25.07.2000 to 19.01.2001.
9. Dr. Shiv Gautam 20.01.2001 to till date

Waiting Hall in the OPD - Psychiatric Center, Jaipur

Psychiatric Center, Jaipur OPD - Reception, Registration and Drug Dispensing at single window

Reception, Psychiatric Center, Jaipur (Front View)

OPD Block, Psychiatric Center, Jaipur (Front View)

Geriatric Care Center, Psychiatric Center, Jaipur

Display at the Rehabilitation Center, Psychiatric Center, Jaipur

Paintings made by patients

Some products made by patients at the rehabilitation center

